# Based on bibliometric visual analysis, the current status and development trends of research on complications after cholecystectomy

**DOI:** 10.3389/fsurg.2025.1586139

**Published:** 2025-05-16

**Authors:** Xiangjie Cheng, Fengchen Hao, Zhan Wang

**Affiliations:** ^1^ Qinghai University, Xining, Qinghai, China; ^2^ Department of Hepatopancreatobiliary Surgery, Affiliated Hospital of Qinghai University, Xining, Qinghai, China; ^3^ Department of Medical Engineering Integration and Translational Application, Affliated Hospital of Qinghai University, Xining, Qinghai, China

**Keywords:** cholecystectomy, complications, bibliometrics, VOSviewer, CiteSpace, bibliometric analysis

## Abstract

**Background:**

Cholecystectomy is a common procedure for treating gallbladder diseases such as cholecystitis and cholelithiasis. Potential complications include bile leakage, infection, bleeding, bile duct injury, and residual bile duct stones, which can significantly affect recovery, quality of life, and overall health. Research on these postoperative complications has gained increasing attention in recent years.

**Objective:**

This study aims to systematically review international literature on cholecystectomy postoperative complications published between 2004 and 2023. The goal is to explore current research trends, hotspots, and developments, providing valuable insights for preventing and managing these complications.

**Results:**

A total of 6,890 articles were retrieved from the WoS database, including 6,173 original research papers and 717 reviews. The publication volume has steadily increased over the past 20 years. The top three countries in publication volume are the U.S., China, and Italy. Sandblom G is the most prolific author, and Harvard University is the most cited institution. The highest volume of publications is in the Surgery field, with Surgical Endoscopy and Other Interventional Techniques being the leading journal. Recent research hotspots include safety, delayed cholecystectomy, guidelines, and postoperative complications.

**Conclusion:**

This bibliometric analysis highlights the steady growth of research on postoperative complications of cholecystectomy since 2004, focusing on complication management, prevention, and laparoscopic cholecystectomy risks. Future research should prioritize improving surgical safety, developing delayed cholecystectomy strategies, and creating clinical guidelines to support ongoing advancements in the field.

## 1 Introduction

Cholecystectomy, as the primary surgical intervention for treating gallbladder diseases such as cholecystitis and cholelithiasis, has been widely applied in clinical practice. With the continuous advancement of minimally invasive techniques, laparoscopic cholecystectomy has become the preferred surgical approach due to its significant advantages, such as minimal trauma and rapid recovery, making it highly favored by both patients and surgeons (1). However, complications such as abdominal pain, bloating, diarrhea, infection, bleeding, and bile leakage remain significant and cannot be overlooked (2, 3). These complications not only severely affect patients’ quality of life but may also pose life-threatening risks. Therefore, in-depth research on the complications of cholecystectomy is of great clinical significance. After cholecystectomy, it is crucial to distinguish between common postoperative pain and pain that arises as a complication. Common postoperative pain typically occurs within a few days after surgery and gradually diminishes over time. This pain is usually localized around the incision and can be controlled with routine analgesics, generally not significantly impacting the patient's daily activities. However, pain associated with complications may be more intense and persistent, often accompanied by symptoms such as fever and nausea. This pain may result from complications such as infection, bleeding, or bile leakage, necessitating imaging and laboratory tests for diagnostic support. Therefore, if a patient experiences unusually severe or persistent pain postoperatively, or if accompanied by other discomforting symptoms, prompt medical evaluation is required to assess and address any potential complications in a timely manner. In the study of complications related to cholecystectomy, scholars have accumulated a large body of literature. To systematically and effectively organize and analyze these publications, a series of specialized bibliometric analysis tools have emerged. Among them, VOSviewer, CiteSpace, and the R package “bibliometrix” have become popular and practical in the field of bibliometric analysis due to their unique functions and advantages, providing new perspectives and methods for research on cholecystectomy complications.

VOS viewer is a Java-based software used for constructing and visualizing bibliometric networks. It generates intuitive visual charts, such as co-citation networks, collaboration networks, and keyword co-occurrence networks, to display the literature structure and research hotspots in a specific discipline or field of knowledge. It is characterized by a user-friendly interface and strong data processing capabilities (4, 5). CiteSpace is a Java-based application for bibliometric analysis and visualization, which constructs knowledge maps to depict the historical progression and research frontiers of scientific literature in a particular discipline or field over a given period. It is known for its simplicity and efficiency (6, 7). The “bibliometrix” package is an R-based bibliometric analysis tool that integrates various statistical analysis and visualization functions, enabling in-depth exploration of the literature data in a specific discipline or field. It reveals research trends and knowledge structures, offering rich statistical analysis and diverse visualization options (8, 9). In this study, we conducted a visual analysis of relevant literature on postoperative complications of cholecystectomy in the Web of Science Core Collection using VOSviewer (v.1.6.20), CiteSpace (v.6.4.R1), and the R package “bibliometrix.” We generated scientific knowledge maps such as cluster analysis and co-occurrence analysis to explore the current status, frontiers, and trends of research, providing a reference for future studies.

## 2 Methods

### 2.1 Data collection

A subject term search was conducted in the Web of Science Core Collection. The search period was from January 2004 to December 2023. The language was English. The search query used was: [TS = (Cholecystectomies OR Cholecystectomy)] AND TS = (Complication OR Complications). The data retrieved was collected on November 10, 2024, to avoid any potential bias due to daily updates. We utilized the built-in deduplication feature of CiteSpace software. After importing the downloaded “download_.txt” file into CiteSpace, we configured the settings under the “Remove Duplicates” option in the Data menu and then ran the deduplication process. CiteSpace automatically identifies and removes duplicate citation records, retaining only unique and relevant references. Inclusion criteria: (1) Relevant to complications after cholecystectomy; (2) Clinical trials, systematic reviews and meta-analyses, animal studies, observational trials, reviews, etc.; (3) Document type: Article or Review Article. Exclusion criteria: Irrelevant documents such as conference proceedings, newspapers, advertisements, letters, etc., and duplicate articles. A total of 6,890 records were obtained. The search results were exported in plain text format with the “Full Record and Cited References” of the retrieved documents, and the file was named “download_.txt.” The total number of citations was 176,702, with 92,928 cited articles and an h-index of 134, with an average of 25.650 citations per item.

### 2.2 Data analysis

**Figure 1:**
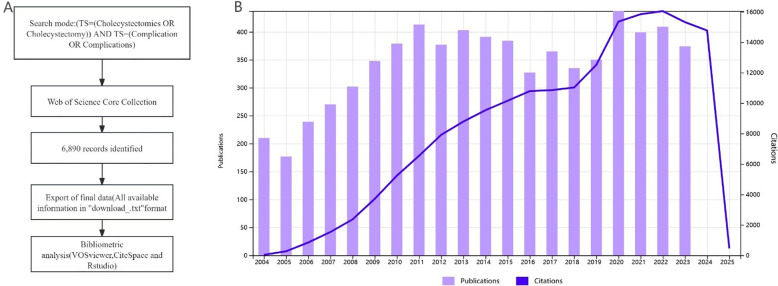
Retrieval strategy, selection process, and publication trends over the last 20 years. **(A)** Retrieval strategy and selection process flowchart. **(B)** Graph of the number of annual publications and citation frequency over the last 20 years.

VOS viewer (v.1.6.20), CiteSpace (v.6.4.R1), and the R package “bibliometrix” were used to analyze all 6,890 documents. VOSviewer, a bibliometric software, is a free, Java-based software developed by Van Eck and Waltman at the Centre for Science and Technology Studies (CWTS) at Leiden University in the Netherlands in 2009. It has strong graphical capabilities and is suitable for handling large-scale data (4). CiteSpace was developed by Professor Chaomei Chen at Drexel University in the United States. It is a document visualization analysis software gradually developed for bibliometric analysis and data visualization (6). “Bibliometrix” is an R-based bibliometric analysis package developed by Aria and Cuccurullo at the University of Padua in Italy in 2017. It provides a rich set of statistical methods and visualization tools, making it suitable for handling large-scale literature data (8). The emergence and development of these three software tools have significantly advanced the research and application expansion in the field of information visualization. Figure 1A shows the flowchart of the search strategy and selection process in this study.

## 3 Results

### 3.1 Temporal distribution map of the literature

Over the past 20 years, the number of publications has shown an overall increasing trend. Since 2009, the number of publications has remained around 350 annually. In 2020, 437 papers were published. The citation frequency began to rise rapidly from 2004 and has maintained a steady upward trend over the past 20 years (Figure 1B).

### 3.2 Distribution of countries/regions

As illustrated in Table 1, the United States, China, and Italy lead in the volume of publications on post-cholecystectomy complications, contributing 24.746%, 10.319%, and 6.662% of the global output, respectively. Collectively, these three countries are responsible for over 40% of all articles, which demonstrates a concentrated research effort and strong academic investment in this domain. Notably, the United Kingdom, the United States, and Italy exhibit the highest average citation rates per article (33.680, 31.770, and 26.950, respectively), suggesting that their research not only has substantial quantity but also considerable influence and maturity within the field. This disparity between publication output and citation impact highlights that some countries are able to produce fewer but highly impactful studies, likely due to more established research infrastructures and strong international collaborations.

**Table 1:** Top 10 countries/regions with the largest number of publications.

Rank	Countrys/Regions	Record count	% Of 6,890	Average per item	H-index	Citations	Total link strength	Centrality
1	USA	1,705	24.746%	31.77	101	54,176	529	0.35
2	China	711	10.319%	14.08	43	10,011	123	0.01
3	Italy	459	6.662%	26.95	54	12,372	372	0.14
4	England	414	6.009%	33.68	55	13,943	302	0.05
5	Japan	396	5.747%	22.23	41	8,803	131	0.00
6	Germany	378	5.486%	25.21	46	9,530	334	0.07
7	Korea	349	5.065%	19.15	42	6,684	89	0.04
8	Turkey	315	4.572%	13.44	33	4,235	32	0.00
9	India	223	3.237%	17.25	34	3,846	129	0.02
10	France	220	3.193%	24.52	40	5,395	244	0.06

Network analysis using betweenness centrality reveals that the United States (0.35) and Italy (0.14) are key nodes in the global research network, underscoring their pivotal role in connecting and driving scientific collaboration. Canada (0.12) also emerges as an important connector. Such centrality indicates that these countries not only contribute significant research but also facilitate cross-border knowledge exchange and help set international research agendas. However, the dominant position of a few high-income countries points to an ongoing imbalance in global scientific contribution. Enhancing participation from underrepresented regions would help diversify research perspectives and improve the generalizability of findings.

**Figure 2:**
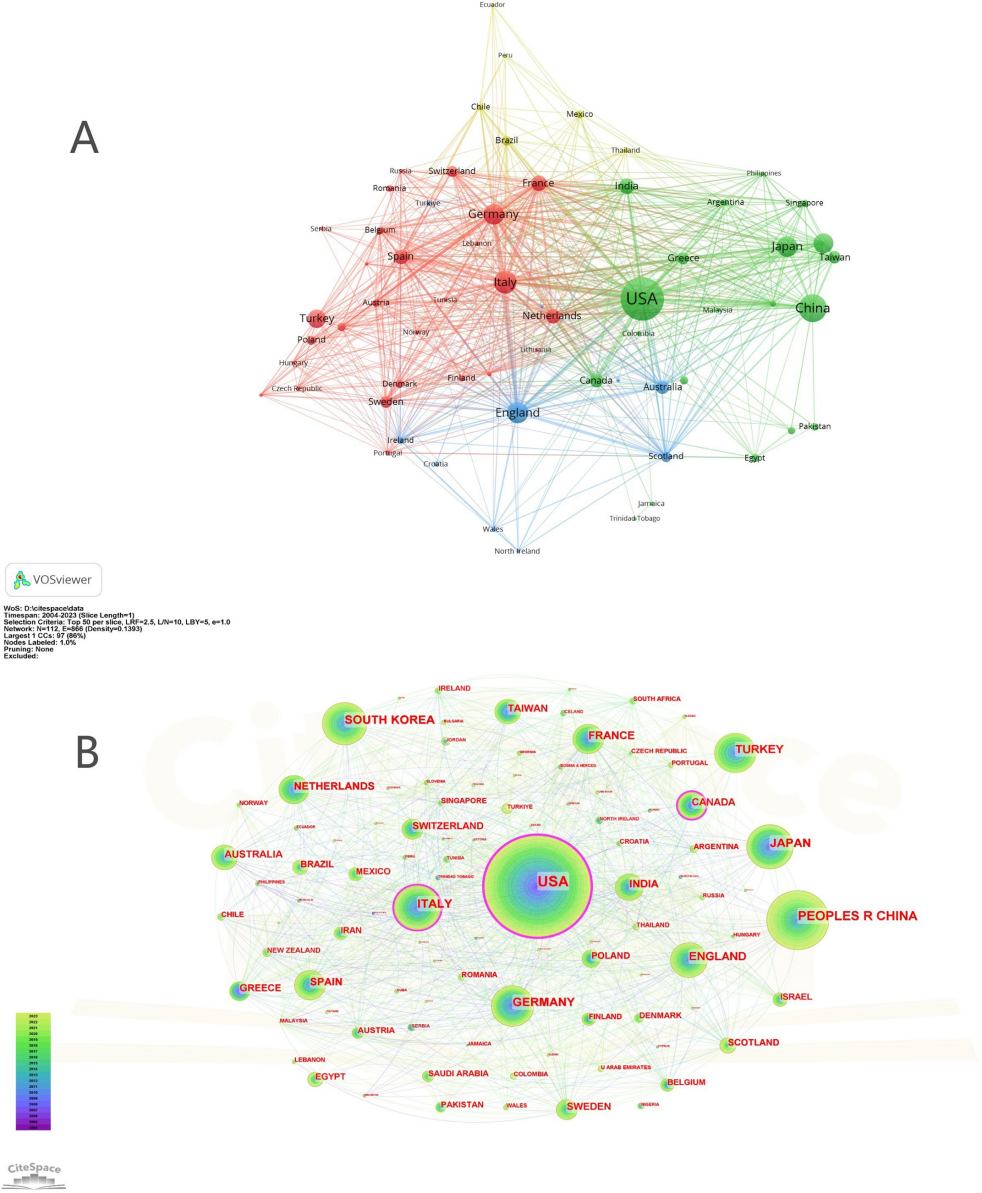
Cooperation map of countries/regions **(A)** A visual map for VOSviewer network. **(B)** A visual map for CiteSpace network.

Figure 2A shows the co-authorship map of countries/regions in the field of post-cholecystectomy complications. Figure 2B shows the visual map for CiteSpace network. The graphical parameters and the specific content of the graphics are provided in the Supplementary Material. As shown in Figure 2A, the global co-authorship network further highlights clusters of regional collaboration, while Figure 2B (visualized with CiteSpace) confirms the influential roles of these central countries. The color gradient from purple to yellow over time in Figure 2B reflects the evolving landscape of international engagement from 2004 to 2023.

### 3.3 Author distribution

Sandblom G from Karolinska Institutet stands out as the most prolific author, followed by Enochsson L from the same institution and Kim JH from Ajou University (Table 2). Notably, the analysis reveals that the betweenness centrality of all authors is zero, indicating an absence of prominent intermediary or bridging figures within the collaboration network. This suggests a decentralized author landscape, where knowledge production is distributed among several groups rather than dominated by a few influential individuals.

**Table 2:** Top 10 authors with the most published papers.

Rank	Author	Record count	% Of 6,890	Affiliations	Average per item	H-index	Centrality
1	Sandblom G	38	0.552%	Karolinska Institutet	15.82	13	0.00
2	Enochsson L	30	0.435%	Karolinska Institutet	17.33	11	0.00
3	Kim JH	27	0.392%	Ajou University	13.67	7	0.00
4	Boerma D	26	0.377%	St Antonius Hospital Utrecht	49.58	17	0.00
5	Davidson BR	26	0.377%	Ucl Medical School	58.78	17	0.00
6	Yang J	26	0.377%	State University Of New York Suny System	21.92	10	0.00
7	Gurusamy KS	25	0.363%	University College London	66.83	23	0.00
8	Gouma DJ	24	0.348%	University Of Amsterdam	100.36	14	0.00
9	Talamini MA	24	0.348%	University Of California San Diego	43	16	0.00
10	Horgan S	23	0.334%	University Of California San Diego	27.04	14	0.00

**Figure 3:**
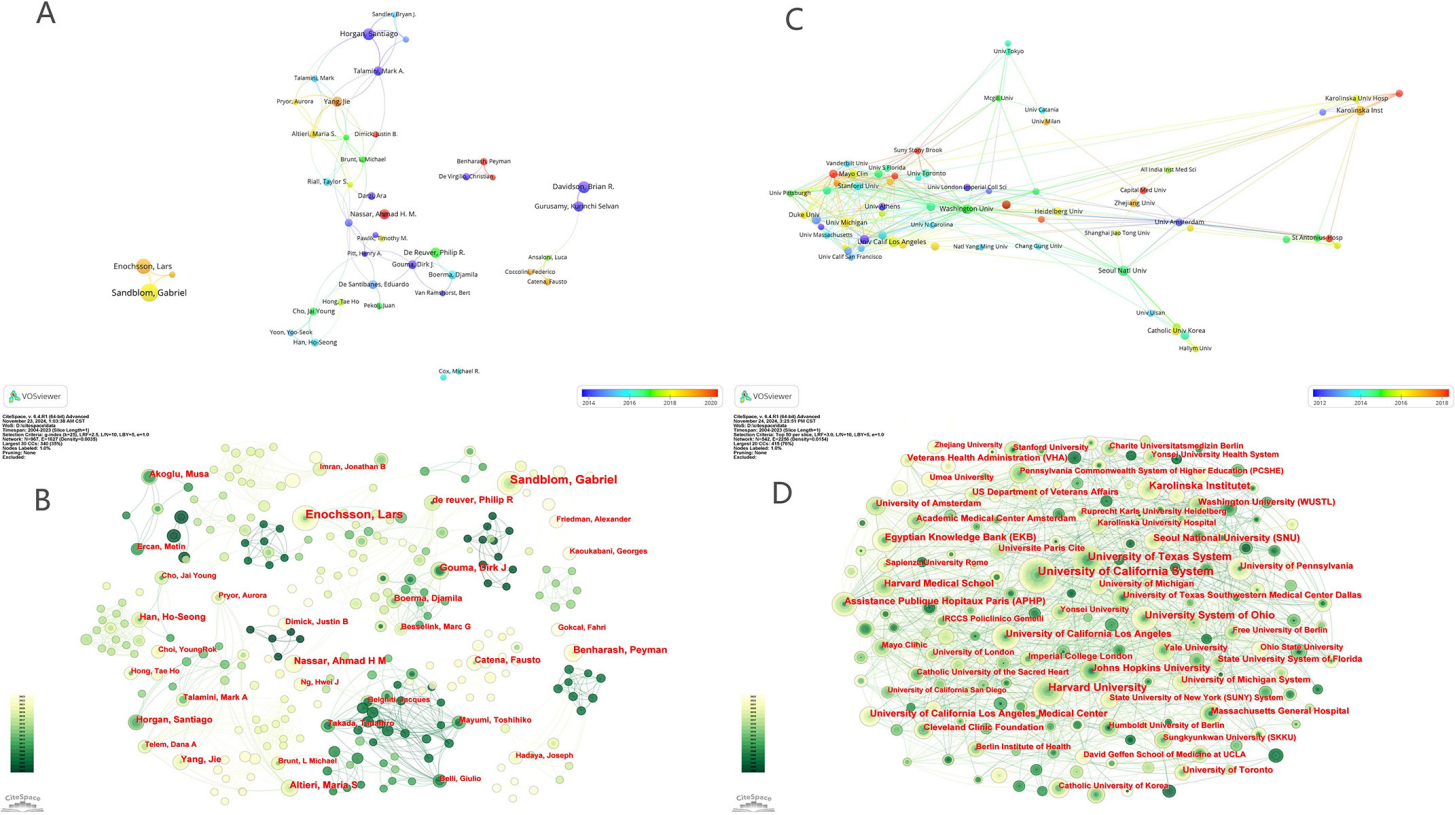
Collaboration networks among authors and among institutions. **(A)** A visual map for VOSviewer network among authors. **(B)** A visual map for CiteSpace network among authors. **(C)** A visual map for VOSviewer network among institutions. **(D)** A visual map for CiteSpace network among institutions.

Figure 3A shows the visual map for VOSviewer network among authors. Figure 3B shows the visual map for CiteSpace network among authors. The graphical parameters and the specific content of the graphics are provided in the Supplementary Material. In Figure 3A, the node size corresponds to each author's publication count, while the lines depict collaborative relationships. The observed clustering pattern indicates that research in this field tends to be conducted within relatively isolated teams or institutional groups, rather than through extensive inter-group or international collaborations. This dispersed structure could foster diversity and independent innovation, but may also hinder the rapid dissemination of novel methodologies or the establishment of field-wide consensus.

Figure 3B, generated with CiteSpace, further confirms the absence of central hubs in the author network. The temporal color gradient shows the evolution of author activity over time, but no author consistently dominates collaboration or output across the entire study period. This is in contrast to some other medical fields, where a small number of key opinion leaders often drive both collaboration and citation impact. Moving forward, encouraging broader and more integrated cooperation—especially cross-institutional and cross-national—may facilitate knowledge sharing and accelerate advances in the study of post-cholecystectomy complications.

### 3.4 Distribution of research institutions

As outlined in Table 3, the University of California System, Harvard University, and the University of Texas System rank as the leading institutions in publication output within the field of post-cholecystectomy complications, contributing 167, 131, and 105 articles, respectively. Johns Hopkins University, Harvard University, and the University of London demonstrate the highest average citation rates per article, indicating not only a strong research presence but also substantial academic impact. This concentration of high-output and high-impact institutions suggests that research efforts and resources remain clustered within a small group of well-established universities, which frequently set the direction for the discipline and influence global academic standards.

**Table 3:** Top 10 organizations in terms of publication volume.

Rank	Institution	Record count	% Of 6,890	Citing articles	Times cited	Average per item	H-index	Centrality
1	University of California System (USA)	167	2.424%	5,350	5,912	35.4	41	0.04
2	Harvard University (USA)	131	1.901%	6,149	6,554	50.03	39	0.08
3	University of Texas System (USA)	105	1.524%	3,451	3,827	36.45	35	0.08
4	University System of Ohio (USA)	81	1.176%	2,329	2,522	31.14	28	0.04
5	Assistance Publique Hopitaux Paris Aphp (France)	77	1.118%	1,978	2,132	27.69	24	0.09
6	Karolinska Institutet (Sweden)	72	1.045%	1,923	2,296	31.89	24	0.02
7	University of California Los Angeles (USA)	70	1.016%	2,029	2,131	30.44	22	0.01
8	Harvard Medical School (USA)	69	1.001%	2,472	2,529	36.65	27	0.01
9	Johns Hopkins University (USA)	66	0.958%	3,150	3,326	50.39	28	0.05
10	University of London (UK)	65	0.943%	2,731	3,074	47.29	32	0.03

Betweenness centrality analysis shows that Hopitaux Paris Aphp, Harvard University, and the University of Texas System are important hubs in the collaborative network, albeit with relatively modest centrality values (0.09 and 0.08). This indicates that, while these institutions play key bridging roles in facilitating collaboration, the overall network is still somewhat fragmented, with many collaborations occurring within distinct regional or institutional clusters. Such a structure may encourage deep specialization within groups, but could also limit broader knowledge exchange and interdisciplinary innovation.

The VOSviewer network visualization (Figure 3C) identifies seven clusters, each representing closely collaborating groups of institutions. Figure 3D (CiteSpace analysis) further highlights the dominance of leading American universities and reveals that the majority of research activity is concentrated in a handful of large nodes. The annual ring widths and color gradients reflect both the temporal and quantitative dimensions of institutional output.

While these patterns are consistent with global trends in medical research—where a few elite institutions often drive much of the scientific progress—the relatively low centrality scores and the presence of multiple clusters indicate that opportunities remain for expanding international and cross-institutional collaborations. Strengthening partnerships with less-represented or emerging institutions worldwide would help diversify perspectives, enhance knowledge transfer, and promote more inclusive and robust research on post-cholecystectomy complications.

### 3.5 Distribution of disciplines and journals

Surgery dominates the research landscape of post-cholecystectomy complications, accounting for 65.138% of all publications, with Gastroenterology & Hepatology (17.228%) and General Internal Medicine (8.258%) following as the next most represented disciplines (Table 4). In addition, fields such as Radiology, Nuclear Medicine, Medical Imaging (2.424%), and Anesthesiology (2.235%) also contribute, reflecting the increasing importance of multidisciplinary approaches to both diagnosis and perioperative management.

**Table 4:** Top 20 subject categories in terms of publication volume.

Rank	Record count	Web of science categories	% Of 6,890
1	4,488	Surgery	65.138%
2	1,187	Gastroenterology Hepatology	17.228%
3	569	Medicine General Internal	8.258%
4	167	Radiology Nuclear Medicine Medical Imaging	2.424%
5	154	Anesthesiology	2.235%
6	147	Medicine Research Experimental	2.134%
7	147	Pediatrics	2.134%
8	117	Obstetrics Gynecology	1.698%
9	101	Oncology	1.466%
10	83	Urology Nephrology	1.205%
11	79	Emergency Medicine	1.147%
12	74	Veterinary Sciences	1.074%
13	68	Pharmacology Pharmacy	0.987%
14	50	Critical Care Medicine	0.726%
15	48	Multidisciplinary Sciences	0.697%
16	43	Clinical Neurology	0.624%
17	41	Transplantation	0.595%
18	39	Health Care Sciences Services	0.566%
19	38	Public Environmental Occupational Health	0.552%
20	37	Hematology	0.537%

Regarding journals, “Surgical Endoscopy and Other Interventional Techniques” leads with the largest number of publications (718 articles), followed by several specialized surgical journals. However, the journals with the highest impact factors among the most cited—such as the “British Journal of Surgery” (IF 8.7) and “Annals of Surgery”—indicate that the most influential research is often disseminated in top-tier, general surgery periodicals (Tables 5, 6). This suggests a balance between technical advancement, as reflected in specialty journals, and broader clinical significance, as seen in higher-impact, more generalist titles.

**Table 5:** Top 10 journals and co-cited journals by publication volume.

Rank	Record count	% Of 6,890	Journal	IF	JCR	Co-cited journal	Frequency	Degree	Centrality	IF	JCR
1	718	10.421%	Surgical Endoscopy And Other Interventional Techniques	2.4 (2023)	Q1	Surgical Endoscopy And Other Interventional Techniques	4,014	55	0.42	2.4 (2023)	Q1
2	246	3.570%	Journal Of Laparoendoscopic & Advanced Surgical Techniques	1.1 (2023)	Q3	Annals Of Surgery	3,386	49	0.14	7.9 (2023)	Q1
3	210	3.048%	Surgical Laparoscopy Endoscopy & Percutaneous Techniques	1.1 (2023)	Q3	British Journal Of Surgery	2,827	47	0.12	8.7 (2023)	Q1
4	137	1.988%	American Surgeon	1.0 (2023)	Q3	American Journal Of Surgery	2,790	44	0.10	2.7 (2023)	Q1
5	132	1.916%	Journal Of Gastrointestinal Surgery	2.2 (2023)	Q3/Q2	Archives Of Surgery	2,371	43	0.07	4.926 (2014)	Q1
6	125	1.814%	World Journal Of Surgery	2.3 (2023)	Q2	World Journal Of Surgery	2,129	40	0.05	2.3 (2023)	Q2
7	118	1.713%	Hepato-Gastroenterology	0.792 (2015)	Q4/Q4	Journal Of The American College Of Surgeons	2,110	34	0.02	3.8 (2023)	Q1
8	117	1.698%	American Journal Of Surgery	2.7 (2023)	Q1	Journal Of Gastrointestinal Surgery	1,898	33	0.03	2.2 (2023)	Q3/Q2
9	109	1.582%	World Journal Of Gastroenterology	4.3 (2023)	Q1	Surgical Endoscopy And Other Interventional Techniques	1,729	28	0.02	2.4 (2023)	Q1
10	104	1.509%	Jsls-Journal Of The Society Of Laparoendoscopic Surgeons	1.4 (2023)	Q3	Surgery	1,728	33	0.06	3.2 (2023)	Q1

**Table 6:** Top 10 cited references of publications.

Rank	Frequency	Centrality	Title	Journal	Author	Year
1	145	0.03	Surgery without scars–Report of transluminal cholecystectomy in a human being	Arch Surg-Chicago	Marescaux J (10)	2007
2	120	0.05	Tokyo Guidelines 2018: flowchart for the management of acute cholecystitis	J Hepato-Bil-Pan Sci	Okamoto K (11)	2018
3	108	0.01	Tokyo Guidelines 2018: diagnostic criteria and severity grading of acute cholecystitis (with videos)	J Hepato-Bil-Pan Sci	Yokoe M (12)	2018
4	102	0.01	Single-incision laparoscopic cholecystectomy: surgery without a visible scar	Surg Endosc	Tacchino R (14)	2009
5	86	0.01	The “invisible cholecystectomy”: A transumbilical laparoscopic operation without a scar	Surg Endosc	Cuesta MA	2008
6	78	0.01	Different pain scores in single transumbilical incision laparoscopic cholecystectomy versus classic laparoscopic cholecystectomy: a randomized controlled trial	Surg Endosc	Tsimoyiannis EC	2010
7	75	0.01	Transcolonic endoscopic cholecystectomy: a NOTES survival study in a porcine model	Gastrointest Endosc	Pai RD	2006
8	73	0.01	Flexible transgastric peritoneoscopy: a novel approach to diagnostic and therapeutic interventions in the peritoneal cavity	Gastrointest Endosc	Kalloo AN	2004
9	71	0.02	Tokyo Guidelines 2018: surgical management of acute cholecystitis: safe steps in laparoscopic cholecystectomy for acute cholecystitis (with videos)	J Hepato-Bil-Pan Sci	Wakabayashi G	2018
10	70	0.12	Transumbilical single-port laparoscopic cholecystectomy	Surg Endosc	Hong TH	2009

**Figure 4:**
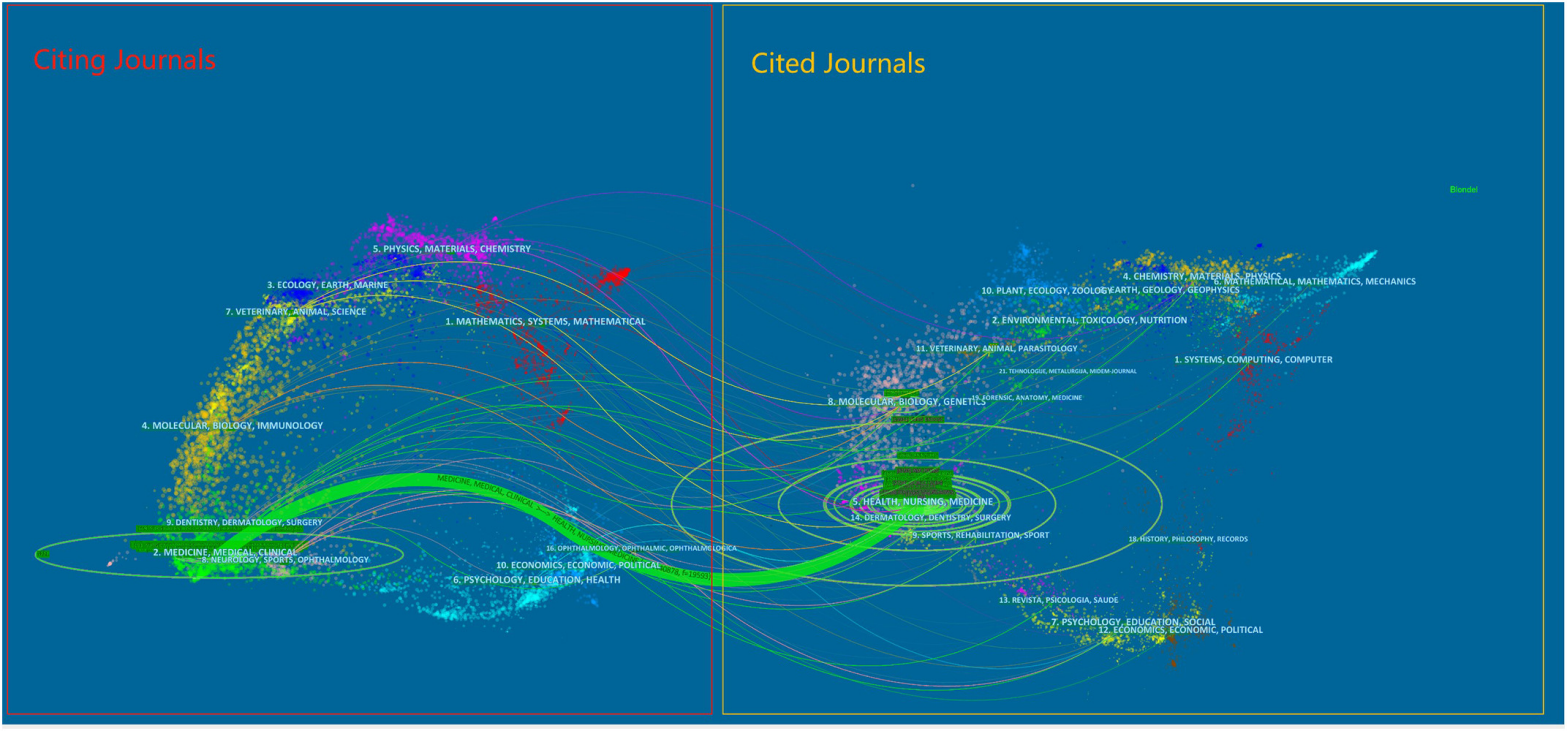
The dual-map overlay of journals.

The dual-map overlay (Figure 4) highlights that research published in health, nursing, and medicine journals serves as a foundational source for studies in clinical medicine and surgery, emphasizing the translational nature of the field. These citation paths reveal strong knowledge integration from related health disciplines into surgical practice. Nevertheless, the limited representation of non-surgical specialties underscores a need for even greater interdisciplinary collaboration, particularly in areas such as perioperative care and long-term outcome evaluation.

Overall, while surgical specialties and journals remain central, expanding collaboration with allied disciplines could enhance innovation and promote more holistic patient care. This multidisciplinary approach will be essential to address the increasingly complex challenges faced in the management of post-cholecystectomy complications.

### 3.6 Co-cited references and references bursts

**Figure 5:**
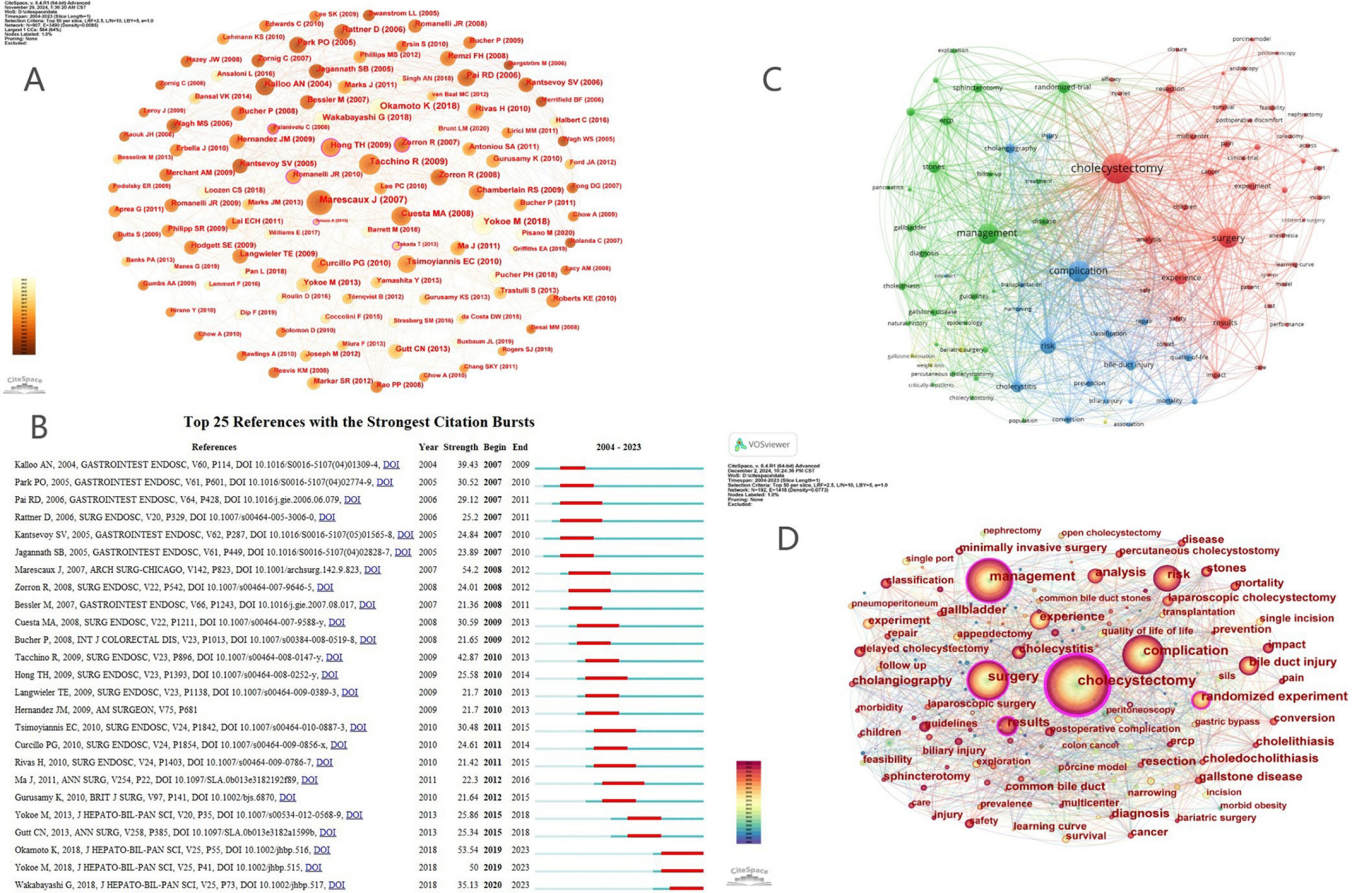
**(A)** References co-citation networ. **(B)** Top 25 references with the strongest citation burst. **(C)** Keyword co-occurrence Network graph based on VOSviewer. **(D)** Keyword co-occurrence Network graph based on CiteSpace.

Figure 5A displays the co-citation network of references, with further graphical details provided in the Supplementary Material. Through CiteSpace analysis, key references with high citation frequency and centrality—such as “Surgery without scars—Report of transluminal cholecystectomy in a human being” (10), the “Tokyo Guidelines 2018” papers (11, 12), the “Transumbilical single-port laparoscopic cholecystectomy” (13) and the “Single-incision laparoscopic cholecystectomy: surgery without a visible scar” (14)—emerge as the intellectual foundation of the field (Tables 6, 7). These landmark articles mark pivotal developments, including the introduction of NOTES, standardized management algorithms for acute cholecystitis, and advances in minimally invasive techniques. The specific content of the literature is summarized in the Supplementary Material.

**Table 7:** Top 20 keywords.

Rank	Keywords	Occurrences	Total link strength	Centrality
1	Cholecystectomy	2,784	8,378	0.33
2	Complication	1,447	4,901	0.08
3	Management	1,370	4,714	0.12
4	Surgery	1,339	3,821	0.16
5	Risk	834	3,165	0.08
6	Bile duct injury	527	1,279	0.04
7	Experience	516	1,717	0.05
8	Results	510	1,821	0.12
9	Randomized experiment	477	1,681	0.10
10	Cholecystitis	390	1,450	0.03
11	Analysis	336	1,170	0.07
12	Stones	334	1,799	0.04
13	Cholelithiasis	299	749	0.05
14	Cholangiography	297	1,378	0.05
15	Sphincterotomy	288	1,396	0.03
16	Ercp	268	1,249	0.03
17	Resection	262	860	0.07
18	Diagnosis	237	778	0.05
19	Disease	226	858	0.04
20	Choledocholithiasis	224	1,075	0.05

Co-citation bursts, as depicted in Figure 5B, further highlight how research attention can rapidly shift in response to technological innovation or guideline updates. The substantial burst intensity associated with Marescaux et al.'s NOTES report (burst intensity 54.2) and the Tokyo Guidelines underscores their transformative impact on surgical practice and academic research. This pattern demonstrates that the field is highly responsive to paradigm-shifting breakthroughs, with citation trends often mirroring major clinical or procedural innovations.

However, the concentration of co-citation around a limited set of high-profile articles also suggests a core-periphery structure, where foundational research strongly guides subsequent work, but may also constrain the recognition of novel or emerging topics. Such a structure enables rapid consensus building and knowledge transfer, yet potentially limits intellectual diversity if less conventional findings are overlooked.

In summary, the current co-citation landscape reflects the field's focus on technological advancement and standardization, driving both scientific inquiry and clinical progress. To sustain innovation, future research should aim to diversify the range of influential works, encourage interdisciplinary approaches, and address broader outcome measures such as patient-reported quality of life and long-term safety.

### 3.7 Research hotspots and frontier analysis

Analysis of keyword frequency and centrality reveals that terms such as “cholecystectomy,” “complication,” “management,” “surgery,” “risk,” and “bile duct injury” have consistently dominated research in this field, underscoring a sustained focus on surgical technique, postoperative management, and especially the critical issue of postoperative complications (Table 7). The frequent appearance of keywords such as “randomized experiment” and “results” further points to a growing emphasis on evidence-based practice and clinical outcomes, reflecting broader trends in surgical research.

The clustering of keywords into four primary themes—surgical techniques and outcomes, management strategies, risk and complications, and metabolic/obesity-related issues—underscores the multidisciplinary nature of post-cholecystectomy complications research. Strong linkages between clusters, as shown in the VOSviewer and CiteSpace network maps (Figures 5C,D), highlight the interconnectedness of technical innovation, clinical management, and evolving disease patterns.

**Figure 6:**
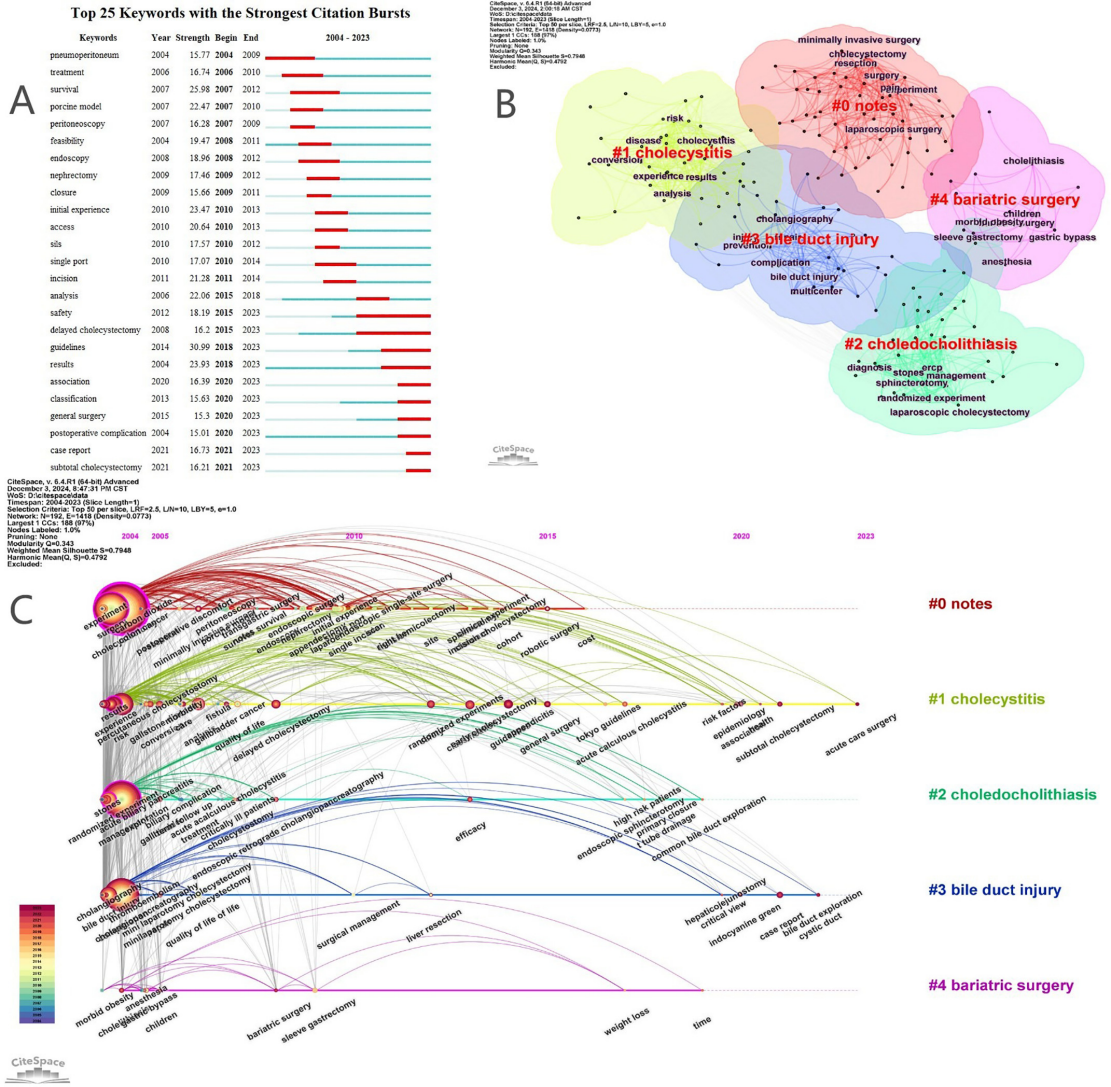
**(A)** Top 25 keywords with the strongest citation bursts. **(B)** Clustering map of keywords. **(C)** Keyword timeline map.

Keyword burst analysis (Figure 6A) reveals dynamic shifts in research priorities, with early interests in experimental and perioperative management giving way to recent surges in safety, delayed cholecystectomy, and clinical guidelines. The emergence of keywords like “SILS,” “endoscopy,” and “feasibility” further reflects ongoing progress in minimally invasive approaches and the increasing pursuit of optimal patient-centered care.

Timeline and cluster analyses (Figures 6B,C) illustrate the transition from traditional open surgery and general complication studies to advanced minimally invasive procedures and a broader evaluation of patient health outcomes. As shown in Table 8, five clusters were formed, namely NOTES, cholecystitis, choledocholithiasis, bile duct injury, and bariatric surgery, numbered from 0 to 4. The clusters with smaller numbers contain more keywords, and each cluster is composed of multiple closely related keywords. However, the relatively limited integration of keywords related to long-term prognosis, psychosocial factors, or health economics suggests areas for future expansion.

**Table 8:** Keyword clustering and related keyword.

Cluster number	Size	Silhouette	Label	LLR clustering keywords
#0	57	0.886	Notes	Notes; single incision; minimally invasive surgery; laparoscopy; laparoscopic surgery
#1	46	0.698	Cholecystitis	Cholecystitis; results; mortality; postoperative complication; percutaneous cholecystostomy
#2	42	0.811	Choledocholithiasis	Choledocholithiasis; ercp; sphincterotomy; common bile duct stones; bile duct injury
#3	30	0.757	Bile duct injury	Bile duct injury; cholangiography; repair; iliary injury; hepaticojejunostomy
#4	13	0.858	Bariatric surjery	Bariatric surjery; gastric bypass; cholelithiasis; sleeve gastrectomy; morbid obesity

**Figure 7:**
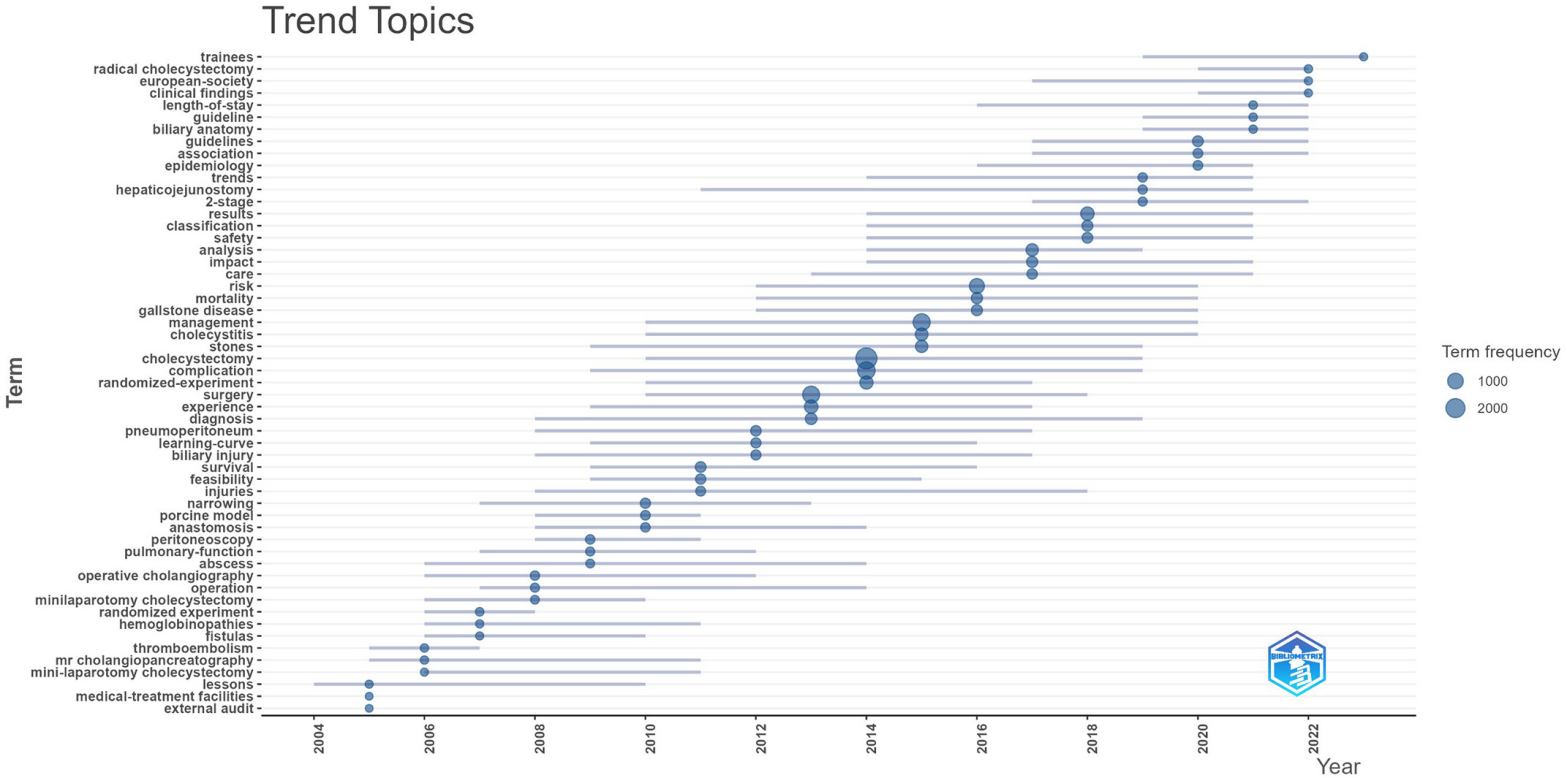
Keyword trend topics graph.

This enduring attention to complications is evident across the field's evolution, as highlighted by the thematic keyword map generated with the R package “bibliometrix” (Figure 7). Over the past two decades, research can be categorized into three stages, each centered around managing postoperative complications: the early stage (2003–2007) focused on open cholecystectomy and the control of common complications; the mid-stage (2008–2013) shifted towards laparoscopic approaches and specific challenges such as bile duct injury and leakage; and the recent stage (2014–present) has seen the expansion of minimally invasive and single-port techniques, alongside increasing attention to the prevention of complications and the improvement of long-term outcomes, including quality of life.

Overall, the persistent and central concern with postoperative complications underscores their importance—not only as a driving force for surgical innovation but also for safeguarding patient health after cholecystectomy. To further advance the field, future research should strengthen cross-disciplinary collaboration, incorporate patient-reported outcomes, and address the wider determinants of postoperative health.

## 4 Discussion

### 4.1 General information

In this study, we utilized CiteSpace, VOSviewer, and Rstudio analytical software to examine the relevant literature in the field of post-cholecystectomy complications. We reviewed the research findings and progress, and conducted a quantitative analysis of basic information, such as annual publication volume, countries, authors, institutions, disciplines, journals, and keywords. Based on the number of publications in the field of post-cholecystectomy complications, the total number of published papers began to rise in 2004, with a cumulative count of 210 papers to date. The higher the citation count of a paper, the greater its impact and quality within the field. As shown in Figure 1B, the citation frequency of these papers has been steadily increasing each year. A statistical analysis of the number of papers published by countries/regions and institutions revealed key countries/regions and research institutions that have published a significant number of influential papers on post-cholecystectomy complications and identified their collaborative relationships. The United States, China, and Italy are the primary countries conducting research on post-cholecystectomy complications. Research in England, the United States, and Italy on post-cholecystectomy complications is relatively advanced. Among the top 10 institutions, 7 are from the United States, with the remaining 3 from France, Sweden, and the United Kingdom. The University of California System is the most prolific institution and has the highest H-index. Collaboration between countries and institutions is notably close, which facilitates the removal of academic barriers and promotes further development in the research of post-cholecystectomy complications.

This sustained increase in publication volume reflects a growing research and clinical interest in post-cholecystectomy complications, likely driven by advances in surgical techniques, the widespread adoption of minimally invasive procedures, and a greater emphasis on patient safety and outcome optimization in recent decades. The plateau in publication numbers since 2009 may suggest the maturation of the field, with foundational research topics well established and newer studies focusing more on refinement and depth rather than sheer volume. The significant rise in citation frequency since 2004 corresponds with periods of technological innovation and the introduction of evidence-based guidelines, which often stimulate both academic output and citation activity. Compared with trends in other surgical research areas, this pattern highlights the global recognition of post-cholecystectomy complications as a critical area requiring continued investigation. Nevertheless, while increased publication and citation rates indicate robust activity, it is important to consider whether this growth is matched by advances in research quality and clinical translation, which should be a focus for future evaluations.

Among the top 10 authors, Sandblom G (38, 0.552%) is the most prolific, followed by Enochsson L (30, 0.435%) and Kim JH (27, 0.392%). This indicates that these three authors have made the most significant contributions to the field of post-cholecystectomy complications. Professor Gurusamy KS from University College London has the highest h-index (15), followed closely by Professor Davidson BR from UCL Medical School, Professor Boerma D from St Antonius Hospital Utrecht, and Professor Talamini MA from University of California San Diego (16–19). The h-index is a comprehensive quantitative metric used to assess the quantity and level of a researcher's academic output. Professors Gurusamy KS and Davidson BR have conducted detailed research into the complexity of post-cholecystectomy complications, analyzing the etiology of gallstones and preventive measures (20), with a particular focus on the safety and efficacy of day-case laparoscopic cholecystectomy (21). Furthermore, Professors Gurusamy KS and Davidson BR also explored the effects of intra-abdominal pressure on post-operative pain and cardiopulmonary complications in laparoscopic cholecystectomy, as well as the safety of low-pressure pneumoperitoneum (22, 23). In the treatment of acute cholecystitis and acute gallstone-induced pancreatitis, the professors compared the advantages and potential risks of early vs. delayed laparoscopic cholecystectomy (16, 24). These research findings not only provide clinical perspectives for understanding the pathogenesis of post-operative complications but also lay an important scientific foundation for the development of more efficient therapeutic strategies. Professor Boerma D studied the incidence of post-cholecystectomy complications and associated factors. Through tracking post-operative symptoms and analyzing medical records, it was found that approximately 14.7% of patients reported colicky pain after cholecystectomy, with most cases occurring within two months as a single event (25). Furthermore, approximately 9.5% of patients required medical treatment for post-operative symptoms and gallstone-related complications, with 2.7% needing acute readmission (26). Professor Boerma D also found that compared to interval cholecystectomy, same-admission cholecystectomy reduced the incidence of recurrent gallstone-related complications in mild gallstone-induced pancreatitis patients, with a very low risk of cholecystectomy-related complications (27). His research highlighted the incidence of post-cholecystectomy colicky pain and suggested possible risk factors and preventive measures, providing clinicians with better communication strategies and tailored surgical recommendations (17). Professor Talamini MA conducted in-depth research into post-cholecystectomy complications. By analyzing extensive medical records and patient follow-up data, Professor Talamini MA found that early cholecystectomy (<72 hours) was associated with a lower rate of complications and bile duct injuries. In a study of 109,862 cholecystectomy cases, patients who underwent early cholecystectomy had fewer complications such as bile duct injury, shorter hospital stays, and lower 30-day readmission and emergency visit rates compared to those who had delayed cholecystectomy (18). Additionally, Professor Talamini MA investigated the delay in cholecystectomy during late pregnancy, finding that although laparoscopic cholecystectomy is safe during pregnancy, it should be considered for delay in late pregnancy due to an increased risk of preterm birth (28). These findings emphasize the importance of early cholecystectomy and provide valuable advice on surgical timing and patient management. Professor Talamini MA's research offers critical guidance for improving treatment outcomes and preventing complications in post-cholecystectomy patients. The centrality of the top 10 authors in terms of publications is 0, with no authors having a centrality ≥0.10, indicating that there are no highly influential authors in this field.

According to the discipline distribution in Table 4, the field of post-cholecystectomy complications has the highest number of articles in the discipline of Surgery (4,488, 65.138%), followed by Gastroenterology Hepatology (1,187, 17.228%) and Medicine General Internal (569, 8.258%). Based on the journal distribution in Table 5, the journal with the most articles in the field of post-cholecystectomy complications is *Surgical Endoscopy and Other Interventional Techniques* (718, 10.421%), followed by *Journal of Laparoendoscopic & Advanced Surgical Techniques* (246, 3.570%) and *Surgical Laparoscopy Endoscopy & Percutaneous Techniques* (210, 3.048%). Three journals are ranked in the Q1 JCR category, with the *World Journal Of Gastroenterology* (4.3, Q1) having the highest impact factor (IF). According to Table 5, the most frequently co-cited journal among the top 10 journals is *Surgical Endoscopy and Other Interventional Techniques*, followed by *Annals of Surgery* and *British Journal of Surgery*. Eight journals are ranked in the Q1 JCR category, and two journals have an IF greater than 5. The highest IF is held by *British Journal Of Surgery* (8.7). The JCR (Journal Citation Reports) overview provides key indicators related to journal impact factors, rankings, and classifications, which are essential for analyzing the sources and distribution of the literature (29). By thoroughly analyzing these data, researchers can identify core journals in the field of post-cholecystectomy complications, thereby providing strong support for the development of scientific achievements. This analysis highlights the significant interest of numerous high-quality and high-impact journals in research related to post-cholecystectomy complications. These data not only offer valuable references for current studies but will also serve as an important basis for future scholars when selecting suitable journals for manuscript submission, further promoting the ongoing development of this field. Figure 4 shows that articles published in journals focused on Health, Nursing, and Medicine are frequently cited by papers in journals related to Medicine, Medical, and Clinical fields. This indicates that current research on post-cholecystectomy complications is mainly concentrated on the practical application in clinical medicine and patient care, as well as interdisciplinary research within the medical domain (30).

The top 10 most-cited references in the publications not only comprehensively cover the latest surgical techniques in the field of cholecystectomy, such as single-port laparoscopic surgery and percutaneous transhepatic cholangioscopy, but also include detailed diagnostic criteria and treatment recommendations for acute cholecystitis from the Tokyo Guidelines 2018 (31–33). The high citation frequency of these references reflects the researchers’ ongoing pursuit of improving surgical efficiency, optimizing post-operative recovery, and emphasizing the importance of standardized clinical management pathways. Current research trends indicate that the treatment of gallbladder diseases is advancing towards more refined, minimally invasive, and standardized approaches (34). Furthermore, this highlights the broad academic recognition and adherence to clinical practice guidelines, which is of significant importance in improving the global treatment level for gallbladder diseases. Looking ahead, with continuous technological innovation and the advancement of clinical research, it is expected that more innovative surgical techniques and treatment strategies will emerge, further promoting the thriving development of the cholecystectomy field.

Based on the high citation burst intensity signal in Figure 5B, it can be inferred that these references had a significant research impact and academic attention during specific time periods. The analysis shows that the citation burst intensity and time intervals of the top 25 references underscore their importance in the field of cholecystectomy (35). The references with the strongest citation bursts indicate that recent research has focused on innovations in surgical techniques, updates to clinical guidelines, and the development of minimally invasive surgeries. Specifically, since 2010, there has been a sharp increase in citations, reflecting the growing attention in the field toward these directions. More specifically, since the early 21st century, researchers have increasingly recognized the importance of combining traditional surgery with minimally invasive techniques. For instance, research into single-port laparoscopic surgery and natural orifice transluminal endoscopic surgery has gained significant attention in recent years, as these techniques greatly reduce surgical trauma and accelerate patient recovery (10, 14, 36, 37). In summary, these highly-cited burst references not only represent research hotspots in the field of cholecystectomy but also signify a critical trend towards transitioning from traditional open surgery to minimally invasive techniques. They provide key evidence for the development and refinement of clinical practices and offer guidance for future research.

Although this study highlights the contributions and academic influence of various countries, institutions, and authors in the field, several critical observations merit further attention. First, while countries like the United States and the United Kingdom demonstrate strong research output and impact, the relatively limited participation of developing countries may reflect disparities in global access to medical research resources. Second, despite some prolific authors having high h-indices, the absence of central figures with high betweenness centrality suggests that the field lacks cohesive leadership or well-established core research teams, with collaboration patterns remaining fragmented. Additionally, most of the highly cited literature focuses on surgical techniques and procedural innovations, indicating a strong emphasis on clinical operability. However, in-depth studies on long-term postoperative complications, quality of life, and patient-specific outcomes remain relatively scarce. Current research themes largely center on “safety, efficiency, and technical innovation,” while personalized treatment strategies and interdisciplinary integration are still in their infancy. Future studies should emphasize multicenter data integration, AI-assisted decision-making systems, and comprehensive postoperative care to guide the evolution of this field toward greater precision, intelligence, and patient-centeredness.

### 4.2 Hotspots and frontiers

Analysis of high-frequency keywords can effectively reflect the hot topics within a specific research field. Through keyword clustering analysis, we identified the main directions and hotspots in the study of post-cholecystectomy complications, revealing the development and evolution of its thematic structure (38). Based on the keyword clustering analysis, five distinct clusters were formed, each represented by a different color. Subsequently, by conducting an in-depth analysis of the top 25 keywords with the strongest citation burst intensity, we further clarified the research hotspots and frontiers in the field of post-cholecystectomy complications. The main findings are as follows:

#### 4.2.1 NOTES and minimally invasive surgical techniques

This clustering study highlights the growing interest in Natural Orifice Transluminal Endoscopic Surgery (NOTES) and minimally invasive techniques, such as single-incision laparoscopic cholecystectomy. NOTES, including transgastric (TG-NOTES), transvaginal (TV-NOTES), and transrectal (TR-NOTES) methods, offer benefits like minimal trauma, faster recovery, and improved postoperative quality of life (39). Professor Judge C's research shows that transvaginal cholecystectomy (TVC) significantly shortens recovery time and enhances functional recovery compared to traditional laparoscopic surgery (40). Additionally, Professor Zhang ZH's study demonstrates that single-incision laparoscopic cholecystectomy (SILC) is comparable to three-port laparoscopic cholecystectomy (TILC) in postoperative outcomes, with reduced pain, and may be preferable for patients with gallstones larger than 2 cm (41). As NOTES technology continues to evolve, its role in cholecystectomy is expected to expand, improving patient care.

#### 4.2.2 Treatment and complications of cholecystitis

This clustering study focuses on cholecystitis treatment, postoperative complications, and percutaneous cholecystostomy drainage. The research findings indicate that effective treatment and prevention strategies for cholecystitis still require further exploration and improvement, which is of great significance for enhancing the safety of cholecystectomy. Professor Jiang H's research demonstrates that combining percutaneous liver puncture cholecystostomy with laparoscopic surgery significantly improves treatment outcomes and reduces complications (42). Professor Niu XY's study confirms that ceftriaxone sodium combined with anti-inflammatory cholagogue tablets is both effective and safe, reducing inflammation and promoting recovery in acute cholecystitis (43). Professor Zhang C's research highlights the advantages of a three-step laparoscopic cholecystectomy for acute refractory cholecystitis, offering superior safety and fewer complications compared to traditional methods (44). Professor Kobayashi S confirms that using endoscopic gallbladder stent placement (EGBS) as an initial treatment, followed by elective cholecystectomy, is safe with minimally invasive surgery (45). As treatment techniques continue to evolve, cholecystitis management is moving toward more refined, minimally invasive, and personalized approaches, offering better outcomes and quality of life for patients. Clinicians must tailor treatment to individual patients to achieve optimal results.

#### 4.2.3 Diagnosis and treatment of choledocholithiasis

This clustering study focuses on the diagnosis and treatment of common bile duct (CBD) stones, including techniques like endoscopic retrograde cholangiopancreatography (ERCP), sphincterotomy, and complications from biliary tract injury. Residual CBD stones are common after cholecystectomy, and their management requires further research. Professor Xiao CH showed that virtual non-contrast (VNC) images from dual-energy computed tomography (CT) provide significant diagnostic value for CBD stones, suggesting dual-energy CT as a promising diagnostic tool (46). Professor Blum J highlighted the potential of machine learning models to assess CBD stone risk, which could help identify patients who might bypass MRCP and proceed directly to intervention, though further validation is needed (47). Professor Han YZ's research indicates that combining ERCP with laparoscopic cholecystectomy is highly effective in treating CBD stones, reducing recurrence, complications, and trauma while improving recovery (48). Professor Weng FZ proposed laparoscopic ultrasound (LUS)-guided CBD exploration and transcystic stone extraction as a safe and effective method, though surgical indications must be carefully considered (49). In summary, advances in diagnostic and treatment techniques for CBD stones, including dual-energy CT, machine learning models, ERCP combined with laparoscopic cholecystectomy, and LUS-guided stone extraction, offer diverse and effective strategies. With ongoing technological advancements and more clinical data, CBD stone management will become more personalized and efficient, improving patient outcomes.

#### 4.2.4 Prevention and treatment of bile duct injury

This clustering study focuses on bile duct injury, including diagnostic methods, treatment approaches (such as cholangiography and repair surgery), and related complications. Bile duct injury, a severe complication after cholecystectomy, requires further optimization in prevention and treatment strategies. Professor Canas-Garcia I's research showed that indocyanine green (ICG) effectively identifies very short cystic ducts in challenging Calot's triangle cases, reducing iatrogenic bile duct injury (50). Professor Stolz MP emphasized that routine use of ICG fluorescence imaging enhances safety by improving visualization of biliary structures and identifying abnormal anatomy, thereby reducing injury risk (51). Professor Yang ZQ highlighted the value of three-dimensional visualization technology for preoperative evaluation and intraoperative navigation, aiding early bile duct injury repair during laparoscopic cholecystectomy (52). Professor Schaub JR's study explored the role of integrins in biliary injury and fibrosis, suggesting that targeting integrins could slow or halt their progression (53).

In summary, bile duct injury prevention and treatment are evolving towards more precise and individualized approaches. With technological advancements and ongoing research, interdisciplinary collaboration and new technologies are expected to reduce injury rates and improve outcomes, offering safer and more effective treatments for patients (54, 55). This research will provide crucial theoretical and technical support for clinical practice, improving bile duct injury management.

#### 4.2.5 Bariatric surgery and gallstones

Bariatric surgery is a key treatment for obesity, but its potential link to gallstone formation and related complications has raised concerns. Studies suggest an association between bariatric surgery and postoperative complications, highlighting the need for further research. Professor Coogan AC's study shows that ursodeoxycholic acid reduces the risk of gallstones, cholecystitis, or cholecystectomy in the first year post-surgery (56). Professor Jiang TX notes that asymptomatic gallstone patients undergoing gastric surgery should typically undergo concomitant cholecystectomy, but preoperative gallbladder evaluation is essential (57). Professor Komorniak N discusses the unclear mechanisms of gallstone formation after gastric bypass, suggesting that gut microbiota and bile acids play key roles (58). Professor Nogueiro J proposes that cholecystectomy should be reserved for symptomatic patients, with factors like high BMI and ultrasound findings for cholesterol stones linked to symptomatic gallstones (59).

In summary, the relationship between bariatric surgery and gallstones is complex. Future research should focus on clarifying the underlying mechanisms, developing preventive strategies, optimizing surgical indications, and providing personalized postoperative care to ensure patient safety and health.

#### 4.2.6 Association, case report and subtotal cholecystectomy are at the forefront of research in this field, currently in a phase of rapid expansion

##### 4.2.6.1 Association

In the current frontier of research, the frequent use of the term “association” clearly reflects researchers’ deep exploration of the potential links between cholecystectomy and other diseases. Future studies will focus on a detailed analysis of related conditions, aiming to identify and explore the interactions between cholecystectomy and key diseases such as osteoporosis, metabolic syndrome, and gastrointestinal disorders (60–62). This objective is aimed at developing more precise, targeted preventive strategies. Additionally, mechanistic research will aim to uncover the fundamental pathophysiological connections between cholecystectomy and various diseases, providing innovative therapeutic insights for both the research community and clinical practice. Moreover, researchers will also focus on developing predictive models based on clinical data, which will integrate patients’ individual characteristics and treatment-related factors, offering solid scientific support for preoperative risk assessment and postoperative management (63).

##### 4.2.6.2 Case report

In the forefront of current medical research, case reports play an indispensable role in revealing the occurrence, progression, and management of post-cholecystectomy complications. By meticulously documenting individual cases, including clinical manifestations, diagnostic processes, and treatment outcomes, case reports provide invaluable empirical data to the medical community (64, 65). Future research will particularly focus on rare and challenging complications, such as postoperative bile duct injury, biliary stricture, and bile leakage. These complications have a profound impact on patients’ quality of life and place higher demands on clinicians’ treatment decisions (66). Studies will explore the etiology, pathophysiological mechanisms, and clinical features of these complex complications, aiming to provide a solid scientific foundation for developing more effective preventive and therapeutic strategies. Additionally, research will emphasize the analysis of innovative treatment methods, including endoscopic surgery, interventional radiology, pharmacological therapies, and organ transplantation, with the goal of improving treatment success rates, reducing complication risks, and enhancing long-term outcomes (67, 68). At the same time, we will actively share comprehensive experiences in managing postoperative complications, including pain control, infection prevention, nutritional support, and psychological counseling, promoting interdisciplinary collaboration and building a holistic complication management network to provide patients with more comprehensive and precise medical care.

##### 4.2.6.3 Subtotal cholecystectomy

In the forefront of current medical research, the indications, surgical techniques, and postoperative complications of subtotal cholecystectomy have become key areas of focus (39). Future research will concentrate on thoroughly investigating the safety and efficacy of this surgical approach (69, 70). Specifically, researchers will conduct comparative analyses between subtotal cholecystectomy and traditional total cholecystectomy to explore whether subtotal cholecystectomy can effectively reduce the risk of postoperative complications and significantly improve patients’ quality of life. Furthermore, studies will examine the long-term outcomes of subtotal cholecystectomy, including postoperative recurrence rates and the likelihood of requiring additional surgeries, in order to clarify its potential advantages and broader prospects for clinical application (71, 72). These findings will provide clinicians with more robust evidence, assisting them in selecting the most appropriate surgical strategy based on the individual circumstances of their patients.

Besides, although the keywords such as Safety, delayed cholecystectomy, guidelines, results, classification, general surgery, and postoperative complications are not brand new, they are currently experiencing a surge in attention. In recent medical research, surgical safety and the prevention of postoperative complications have become central concerns. Minimizing surgical trauma and reducing the risk of complications is crucial, with future studies expected to focus on innovations such as single-incision laparoscopic cholecystectomy and natural orifice transluminal endoscopic surgery (NOTES) (31, 39). These techniques aim to lessen the invasiveness of surgery, thereby lowering complication rates. Moreover, the development of accurate surgical risk assessment models will enable personalized treatment plans, enhancing surgical safety. Postoperative management strategies, including pain control and infection prevention, will also be a primary research focus, with the goal of reducing complications and improving patients’ quality of life.

Another significant area of research is the timing and efficacy of delayed cholecystectomy. As this approach continues to garner attention, future studies will explore the optimal timing for surgery in different patient populations, such as those with acute cholecystitis or choledocholithiasis, as well as its long-term outcomes and safety (73, 74). The effects of delayed surgery on patients’ quality of life and psychological well-being will also be investigated, with a focus on improving patient prognosis.

In the management of post-cholecystectomy complications, clinical guidelines play an increasingly prominent role. Future research will aim to develop more comprehensive and standardized guidelines that address a variety of complications and provide detailed protocols for postoperative care. Ensuring that clinicians adopt and adhere to these guidelines will be essential to improve outcomes. Additionally, the mechanisms for updating these guidelines to maintain their relevance and effectiveness will be a key research direction (75, 76).

The concept of “outcomes” in post-cholecystectomy research highlights the importance of evaluating the long-term effects of surgery. Future studies will focus on long-term follow-up to assess the safety and efficacy of the procedure, identify potential late complications, and examine the overall impact on patients’ health and functionality. Additionally, cost-effectiveness analyses will be integral to evaluate the economic benefits of various treatment strategies, providing evidence to guide decision-making in resource-limited settings (77–79).

The Clavien-Dindo classification system has become a crucial tool in classifying post-cholecystectomy complications. Ongoing research aims to refine this system by considering additional factors such as the timing of complications and underlying pathophysiological mechanisms (80, 81). This will enable more accurate diagnoses and better-tailored treatment plans. Future studies will explore treatment strategies for each classification level, including pharmacological and surgical interventions, to improve therapeutic outcomes (82, 83).

Collaboration between general surgery and other medical fields, such as gastroenterology, endocrinology, and anesthesiology, will also strengthen in the future. These interdisciplinary efforts will enhance the prevention and management of post-cholecystectomy complications, particularly in areas such as choledocholithiasis, diabetes, and the impact of anesthetic drugs (84, 85).

Finally, postoperative complication management remains a critical area of research. Preventive strategies, including pharmacological interventions and dietary adjustments, will be explored to reduce the risk of complications. The development of more precise diagnostic tools, such as imaging and laboratory testing, will enable timely identification and treatment of complications (86, 87). Treatment protocols will also continue to evolve, improving outcomes for conditions such as bile leaks and infections (88, 89). Through these measures, patient health and safety will be significantly enhanced.

### 4.3 Advantages and limitations

This study is the first to apply bibliometric methods to the field of post-cholecystectomy complications, providing a comprehensive and in-depth review and guidance for researchers in this area. By deeply mining extensive literature data, we successfully identified key trends, research patterns, and knowledge gaps in the field, offering clear direction for future research and clinical practice. The study employed three bibliometric tools—VOSviewer, CiteSpace, and the R package “bibliometrix”—to comprehensively analyze the current state and development trends of the field from multiple dimensions.

VOSviewer, through constructing visual network maps, revealed the complex relationships between countries, authors, institutions, journals, and keywords, identifying the major research collaboration networks and key topics. CiteSpace, by creating knowledge maps, demonstrated the trajectory of research hotspots over time, identifying emerging fields and research frontiers. The R package “bibliometrix” provided rich statistical analysis and visualization capabilities, allowing us to delve into the literature data and uncover research trends and the knowledge structure (90).

Compared to traditional literature reviews, bibliometric analysis provides a more comprehensive and detailed insight into the hotspots and frontiers of post-cholecystectomy complications research. Traditional reviews often rely on the manual selection and interpretation of a limited number of papers, which may introduce bias and constrain the scope of understanding. Bibliometric analysis, on the other hand, allows for a broader and more systematic survey, offering a more holistic view of the knowledge landscape, emerging trends, and potential future research directions (91). This comprehensive perspective helps to identify knowledge gaps, highlight areas requiring further investigation, and provide more reliable scientific evidence for clinical practice.

Although this study employed multiple bibliometric tools to conduct a systematic analysis of research on postoperative complications following cholecystectomy and yielded valuable insights, several limitations remain and warrant further consideration in future research.

First, the data sources in this study were restricted to the Web of Science database, which may not comprehensively reflect the entire research landscape in this field. Other scientific databases such as PubMed, Embase, and the Cochrane Library, as well as non-English-language journals, may include additional high-quality and relevant studies that were not captured in the current analysis (92). Second, this study only included English-language publications, which may have resulted in the omission of important findings published in non-English literature. For example, countries such as China, Japan, and South Korea have made substantial contributions to the field of gallbladder disease; however, these findings are often published in native-language journals and thus may not have been retrieved in our search. In addition, this analysis only included studies published up to December 31, 2023, which may not fully represent the most recent developments in the field. Some critical research may have been published after this date, and emerging research topics may not yet have reached sufficient visibility or maturity to be identified through bibliometric analysis.

Regarding the limitations inherent to bibliometric tools themselves, each platform possesses specific strengths and weaknesses that may influence the completeness and accuracy of the results. Specifically, VOSviewer performs well in visualizing bibliometric networks and clearly depicts co-occurrence relationships among keywords, authors, and journals. However, its visualization approach is relatively uniform, relying primarily on density- and distance-based layout algorithms. This may lead to the marginalization of certain key nodes in the network, making it difficult to highlight important research hotspots within the discipline. CiteSpace offers strengths in identifying research trends and tracing the evolution of knowledge domains through citation analysis. However, its visualization capability may be compromised when handling large-scale datasets. As the volume of included literature increases, the generated network maps may become overly complex and crowded, hindering the clarity of inter-nodal relationships—particularly in the context of more intricate research structures. Bibliometrix provides a wide range of analytical functionalities, including collaboration network analysis, co-citation analysis, and keyword trend analysis. Nevertheless, one of its major limitations is that it only supports the analysis of English-language literature, which restricts its applicability to multilingual bibliometric studies. Furthermore, the process of importing R packages and conducting data preprocessing in Bibliometrix can be time-consuming, potentially affecting the overall efficiency of bibliometric analyses, especially when working with large datasets (91). Therefore, in this study, we integrated the strengths and compensated for the weaknesses of different bibliometric tools to conduct a more comprehensive and balanced analysis.

## 5 Conclusions

Through a detailed bibliometric analysis of post-cholecystectomy complications, this study evaluated the literature across different years, countries, institutions, authors, disciplines, and journals, and analyzed the evolution of research themes and future research hotspots. Our study observed that this field has gained attention since 2004, with a steady growth trend. This research provides foundational information on studies in the field and identifies potential collaborators for interested researchers. Current research hotspots primarily focus on the management of gallstone-related complications, post-laparoscopic cholecystectomy complications, and risk factors for gallstone complications. Presently, key research frontiers in this field include safety, delayed cholecystectomy, guidelines, results, association, classification, general surgery, postoperative complications, case reports, and subtotal cholecystectomy, all of which are in an emerging phase.

## Data Availability

The original contributions presented in the study are included in the article/Supplementary Material, further inquiries can be directed to the corresponding author.
