# Supplementary material for: Based on bibliometric visual analysis, the current status and development trends of research on complications after cholecystectomy
Source: Front Surg. 2025 May 16;12:1586139. doi: 10.3389/fsurg.2025.1586139 (PMC12122503; doi:10.3389/fsurg.2025.1586139)
Supplement: Supplementary file 2 [file Datasheet1.pdf]

Supplementary file

Figure 2A parameters: The VOS viewer parameters are set as follows: the algorithm for calculating association strength (Method) is set to "Layout/Clustering Algorithm (Linlog/modularity)," and the minimum number of documents for a country/region (Minimum number of documents of a country) is set to "5." The results obtained are from 113 countries/regions, with 65 countries/regions meeting the threshold. Based on co-authorship analysis by country/region, VOSviewer divides countries/regions into different clusters. Nodes of different colors in the figure represent different clusters. The size of the nodes indicates the number of documents, and the thickness of the lines represents the number of connections between the nodes.

The specific content of Figure 2A: Figure 2A reveals that the United States primarily collaborates closely with China, Taiwan, Canada, Japan, India, Greece, Malaysia, and Colombia; England mainly collaborates with Ireland, Croatia, Scotland, Australia, Wales, Turkey, and Northern Ireland; Italy works closely with Germany, the Netherlands, France, Spain, Turkey, Switzerland, Sweden, and Denmark; Brazil often collaborates with Mexico, Chile, Thailand, Peru, and Ecuador.

Figure 2B parameters: The Cite Space parameters are as follows: the time

slicing (Time Slicing) is set from "January 2004 to December 2023," the years per slice (Years Per Slice) is set to "1," the Term Source is set to "Whole Selection," the node type (Node Types) is set to "Country," and the node selection criteria (Selection Criteria) is set to "Top N=50," with other parameters remaining at their default settings.

Figure 3A parameters： The VOS viewer parameters are set as follows: the algorithm for calculating association strength (Method) is set to "Layout/Clustering Algorithm (Linlog/modularity)," and the minimum number of documents for an author (Minimum number of documents of an author) is set to "10." A total of 31,685 authors were retrieved, with 48 authors meeting the threshold. Based on co-authorship analysis, VOSviewer divides the authors into different clusters and colors them according to their chronological order, seamlessly integrating the time dimension into the author co-occurrence network.

The specific content of Figure 3A： As shown in Figure 3A, different clusters represent the collaborative relationships among authors. Horgan, Santiago, and Jacobsen, Garth R., Talamini, Mark A., and Sandler, Bryan J. collaborate closely; Yang, Jie collaborates closely with Pryor, Aurora, Altieri, Maria S., Dimick, Justin B., Brunt, L. Michael, Riall, Taylor S., Darzi, Ara, Telem, Dana A., Pucher, Philip H., and Talamini, Mark; De

Reuver, Philip R. works closely with Gouma, Dirk J., Boerma, Djamila, Van Ramshorst, Bert, Strasberg, Steven M., Pawlik, Timothy M., Soper, Nathaniel J., Pitt, Henry A., De Santibanes, Eduardo, and Pekolj, Juan; Cho, Jai Young worked closely with Hong, Tae Ho, Yoon, Yoo-Seok, and Han, Ho-Seong; Enochsson, Lars collaborates closely with Sandblom, Gabriel and Osterberg, Johanna; Benharash, Peyman collaborates closely with De Virgilio, Christian and Sanaiha, Yas; Cox, Michael R. works closely with Eslick, Guy D.; Davidson, Brian R. collaborates closely with Gurusamy, Kurinchi Selvan, Ansaloni, Luca, Coccolini, Federico, and Catena, Fausto.

Figure 3B parameters： The CiteSpace parameters are set as follows: the time slicing (Time Slicing) is set from "January 2004 to December 2023," the years per slice (Years Per Slice) is set to "1," the Term Source is set to "Whole Selection," the node type (Node Types) is set to "Author," and the node selection criteria (Selection Criteria) is set to the "g-index option, k=25," with other parameters remaining at their default settings.

Figure 3C parameters： The VOS viewer parameters are set as follows: the algorithm for calculating association strength (Method) is set to "Layout/Clustering Algorithm (Linlog/modularity)," and the minimum number of documents for an organization (Minimum number of documents of an organization) is set to "20." The results were retrieved from 5,901

organizations, with 72 organizations meeting the threshold.

The specific content of Figure 3C： Group 1 consists of the following institutions: Vanderbilt University, Mayo Clinic, Stanford University, Ohio State University, Emory University, Yale University, University of Pittsburgh, University of Alabama at Birmingham, University of Athens, Duke University, Johns Hopkins University, University of Rome La Sapienza, and Shandong University;Group 2 consists of: Harvard University, Harvard Medical School, Cleveland Clinic, Massachusetts General Hospital, University of Illinois, University of Massachusetts, University of California San Diego, Tufts University, China Medical University, and National Yang Ming University;Group 3 consists of: University of Michigan, University of Colorado, University of California San Francisco, Northwestern University, University of California Los Angeles, University of Pennsylvania, SUNY Stony Brook, University of Toronto, Washington University, University of North Carolina, University of South Florida, and University of Arizona;Group 4 consists of: University of Tokyo, Hokkaido University, McGill University, University of Catania, University of Milan, Imperial College London, and Catholic University of the Sacred Heart;Group 5 consists of: Karolinska Institute, Karolinska University Hospital, Umeå University, and University Hospital;Group 6 consists of: University of Amsterdam, University of

Melbourne, University Hospital, Umeå University, and University Hospital;Group 7 consists of: Heidelberg University, Huazhong University of Science and Technology, Chang Gung University, All India Institute of Medical Sciences, Capital Medical University, Zhejiang University, Shanghai Jiao Tong University, Seoul National University, University of Ulsan, Sungkyunkwan University, Catholic University of Korea, Yonsei University, and Hallym University.

Figure 3D parameters：The Cite Space parameters are set as follows: the time slicing (Time Slicing) is set from "January 2004 to December 2023," with "1" year per slice (Years Per Slice), Term Source set to "Whole Selection," node type (Node Types) set to "Institution," and the node selection criteria (Selection Criteria) set to "Top N=50," with other parameters remaining at their default settings.

Figure 5A parameters：The Cite Space parameters are set as follows: Time Slicing is set from January 2004 to December 2023, with each slice representing one year (Years Per Slice = 1). The node type (Node Types) is set to "Cited Reference," and the selection criterion (Selection Criteria) is "Top N=50," with other parameters left at their default settings. Based on these parameters, a network map was generated with 907 nodes, 3,490 connections between nodes, and a network density of 0.0085

Figure 5C parameters： The VOS viewer parameters were set as follows: the algorithm for calculating the association strength (Method) was set to "Layout/Clustering algorithm (Linlog/modularity)," and the minimum number of occurrences of a keyword was set to "50." A total of 5,983 institution-based results were retrieved, with 95 keywords meeting the threshold. For these 95 keywords, the total co-occurrence with other keywords was calculated, which is referred to as the total link strength. The total link strength reflects the degree of association between a given keyword and other keywords. Nodes with higher total link strength typically indicate greater centrality or connectivity within the dataset. The keyword with the highest total link strength is cholecystectomy.

The specific content of Figure 5C： The red cluster mainly includes terms such as cholecystectomy, surgery, results, experience, resection, porcine model, closure, peritoneoscopy, endoscopy, efficacy, injuries, survival, feasibility, nephrectomy, postoperative discomfort, multicenter, pain, colectomy, access, SILS, clinical trial, cancer, port, experiment, children, incision, colorectal surgery, analysis, anesthesia, safety, system, patient, model, cost, impact, care, and performance.

The green cluster primarily consists of management, stones, randomized trial, sphincterotomy, ERCP, exploration, follow-up, treatment,

pancreatitis, gallbladder, disease, diagnosis, cholelithiasis, guidelines, gallstone disease, natural history, epidemiology, cholecystostomy, population, percutaneous cholecystostomy, and critically ill patients.

The blue cluster encompasses complications, risk, cholecystitis, injury, cholangiography, transplantation, narrowing, repair, classification, bile duct injury, quality of life, prevention, biliary injury, mortality, conversion, bile duct, and association.

The yellow cluster mainly includes terms such as gallstone formation, bariatric surgery, weight loss, and obesity.

Figure 5D parameters： The parameters for CiteSpace are set as follows: Time Slicing is set from "January 2004 to December 2023," with the Years Per Slice parameter set to "1." The Node Types are set to "Keywords," and the Selection Criteria is set to "Top N=50," with all other parameters retained at their default settings. Based on these parameters, a network map was generated with 192 nodes, 1418 connections between nodes, and a network density of 0.0773.

3.6 Summary of the specific content of the literature：

The article "Surgery without scars - Report of transluminal cholecystectomy in a human being" by Marescaux J, published in 2007 in Arch Surg-Chicago, primarily introduces natural orifice transluminal

endoscopic surgery (NOTES) as a means to achieve scarless surgery, marking a revolutionary change in surgical techniques. IRCAD-EITS has actively participated in the development of NOTES since 2004 and has established a specialized program for this. A transvaginal NOTES cholecystectomy was performed on a 30-year-old female patient by a multidisciplinary team using standard endoscopic instruments, with no laparoscopic assistance, strictly following the principles of cholecystectomy. The patient experienced no pain, no scarring, and was discharged the next day. NOTES surgery is feasible and safe, offering great benefits to patients, with fewer postoperative complications, and could potentially be the next evolution in surgical procedures.

The article "Tokyo Guidelines 2018: flowchart for the management of acute cholecystitis" by Okamoto K, published in 2018 in J Hepato-Bil-Pan Sci, presents a new treatment flowchart for acute cholecystitis based on the 2018 Tokyo Guidelines. The guidelines recommend that for patients with grade III acute cholecystitis, laparoscopic cholecystectomy may be performed in high-level centers with experienced surgeons, provided strict criteria are met. For grade I and II acute cholecystitis patients, early laparoscopic cholecystectomy is recommended based on their comorbidity index and physical status. If early surgery is not feasible, bile drainage should be performed first, followed by laparoscopic cholecystectomy once the patient’s condition improves. The 2018 Tokyo Guidelines provide clear

guidance on the management of acute cholecystitis, emphasizing the selection of appropriate surgical timing and method based on disease severity and patient condition to improve treatment outcomes, enhance quality of life, and reduce postoperative complications.

The article "Single-incision laparoscopic cholecystectomy: surgery without a visible scar" by Tacchino R, published in 2009 in Surg Endosc, introduces laparoscopic cholecystectomy as the gold standard for gallbladder surgery since 1992. Based on this, the author presents single-incision laparoscopic cholecystectomy (SILS) as a further step in reducing surgical invasiveness. This procedure involves a single 12-mm incision at the umbilicus, with the Veress needle used to induce pneumoperitoneum, followed by insertion of a trocar for the procedure. In the study, 12 patients underwent SILS cholecystectomy without major complications. Among them, 8 had a history of other laparoscopic surgeries, and 3 had a body mass index greater than 35. The operative time was significantly reduced from an initial 3 hours to approximately 50 minutes. The author concluded that SILS cholecystectomy is not only feasible and safe but also effective in minimizing surgical trauma and reducing postoperative complications.

The article "Transumbilical single-port laparoscopic cholecystectomy" has a centrality of 0.12, indicating its significant influence. This study, published in 2009 by Hong TH et al. in Surg Endosc, investigates the use of transumbilical single-port laparoscopic cholecystectomy (TUSPLC) for

the treatment of 15 patients with cholelithiasis, aiming to reduce incisions and improve cosmetic outcomes. The procedure employed a unique "single port" technique, leaving almost no visible scars, and utilized an ultra-small wound retractor and surgical gloves as the single-port channel. None of the cases required additional skin incisions or conversion to standard laparoscopic surgery. The average operative time was 79 minutes, and the average postoperative hospital stay was 1.6 days, with no postoperative complications reported. The study suggests that TUSPLC is an effective and promising scarless abdominal surgery alternative for selected symptomatic cholelithiasis patients. Mastery of key articles in this field contributes to a deeper understanding of the overall research landscape.
